# Decreasing Mortality in Severe Sepsis and Septic Shock Patients by Implementing a Sepsis Bundle in a Hospital Setting

**DOI:** 10.1371/journal.pone.0026790

**Published:** 2011-11-03

**Authors:** Sandra Christina Pereira Lima Shiramizo, Alexandre R. Marra, Marcelino S. Durão, Ângela T. Paes, Michael B. Edmond, Oscar Fernando Pavão dos Santos

**Affiliations:** 1 Critical Care Unit, Hospital Israelita Albert Einstein, São Paulo, Brazil; 2 Statistics Department, Instituto Israelita de Ensino e Pesquisa (IIEP), São Paulo, Brazil; 3 Department of Internal Medicine, Virginia Commonwealth University School of Medicine, Richmond, Virginia, United States of America; Oregon Health and Science University, United States of America

## Abstract

**Background:**

The Surviving Sepsis Campaign (SSC) guidelines for the management of severe sepsis (SS) and septic shock (SSh) have been recommended to reduce morbidity and mortality.

**Materials and Methods:**

A quasi-experimental study was conducted in a medical-surgical ICU. Multiple interventions to optimize SS and SSh shock patients' clinical outcomes were performed by applying sepsis bundles (6- and 24-hour) in May 2006. We compared bundle compliance and patient outcomes before (July 2005-April 2006) and after (May 2006-December 2009) implementation of the interventions.

**Results:**

A total of 564 SS and SSh patients were identified. Prior to the intervention, compliance with the 6 hour-sepsis resuscitation bundle was only 6%. After the intervention, compliance was as follows: 8.2% from May to December 2006, 9.3% in 2007, 21.1% in 2008 and 13.7% in 2009. For the 24 hour-management bundle, baseline compliance was 15.0%. After the intervention, compliance was 15.1% from May to December 2006, 21.4% in 2007, 27.8% in 2008 and 44.4% in 2009. The in-hospital mortality was 54.0% from July 2005 to April 2006, 41.1% from May to December 2006, 39.3% in 2007, 41.4% in 2008 and 16.2% in 2009.

**Conclusion:**

These results suggest reducing SS and SSh patient mortality is a complex process that involves multiple performance measures and interventions.

## Introduction

Severe sepsis and septic shock are the major causes of admission and death in intensive care units (ICUs). The sepsis syndromes are lethal and expensive conditions, with hospital mortality rates for severe sepsis ranging between 30% and 50% [1], [2]. In the United States, this results in an estimated 751,000 cases and 215,000 deaths annually [1]. In Brazil, the incidence density is 57 per 1,000 patient-days and the mortality rate of patients with severe sepsis and septic shock is 47.3% and 52.2%, respectively [3].

In 2004, the Surviving Sepsis Campaign (SSC) introduced guidelines for the management of severe sepsis and septic shock, as well as strategies for bedside implementation [4], [5]. The treatment recommendations were organized in two bundles: a resuscitation bundle (6 tasks to begin immediately and to be accomplished within 6 hours) and a management bundle (4 tasks to be completed within 24 hours). The 6-hour resuscitation bundle includes the lactate determination, early cultures and antibiotic therapy as soon as possible, and “early goal directed therapy” (EGDT) [2]. The first 24-hour management bundle includes optimization of glycemic control, respiratory inspiratory plateau pressure, and determination of the need for corticosteroids and drotrecogin alfa (activated). EGDT is simply a protocol derived from components that have long been recommended as standard care for the septic patient to optimize hemodynamics. Of note, we did not use packed red blood cells as a resuscitation fluid nor did we use dobutamine as a standard of care for all septic shock patients.

The aims of this study were to determine the rate of compliance with 6-hour and 24-hour sepsis bundles, and to determine the impact of compliance on hospital mortality in patients with severe sepsis and septic shock.

## Methods

This study was conducted in the ICU of a tertiary care, private hospital in São Paulo, Brazil. This open model ICU is a 38-bed medical-surgical unit where approximately 2,200 patients are admitted each year.

This was a prospective quasi-experimental, before and after study, comparing time periods before (July 2005 – April 2006) and after (May 2006 – December 2009) implementation of the interventions. An educational program based on the SSC guidelines was implemented in April 2006. We have developed lectures, e-learnings and protocols. After that sepsis bundles were applied for severe sepsis and septic shock patients in our hospital. This education program is reinforced each year through the Continuing Medical Education (CME) in our hospital. The sepsis program is addressed to the healthcare workers where are discussed the social impact of sepsis, the diagnostic and therapeutic interventions, the quality indicators and the process of data collection.

Patients over 18 years old with severe sepsis and septic shock were included in the study. This study was a quality improvement study that was approved by Institutional Review Board (IRB) from Hospital Israelita Albert Einstein. The requirements for informed consent was waived by our IRB in accordance of the Code of Federal Regulations and of the Privacy Rule.

The data collected included age, sex, admission date, the time when severe sepsis or septic shock was diagnosed, location before ICU admission, hospital and ICU length of stay, organ dysfunction at the time of diagnosis, APACHE II score, and outcome status. As per the SSC “time zero” was defined as the time of diagnosis of severe sepsis and septic shock diagnosis.

Once a patient meets the bundle initiation criteria, the 6-hour bundle is initiated by collecting serum lactate and obtaining blood cultures before antibiotic administration. From the time of severe sepsis (time zero), broad-spectrum antibiotic are to be administered within 1 hour. Hypotension and/or elevated lactate are treated with IV fluids; in the event of persistent hypotension despite fluid resuscitation (septic shock) and/or lactate >4 mmol/L (>36 mg/dL), maintaining adequate central venous pressure and central venous oxygen saturation are indicated. Patients who do not have septic shock and elevated lactate >4 mmol/L (>36 mg/dL) do not require measurement of central venous pressure and central venous oxygen saturation.

The 24-hour sepsis bundle for patients with severe sepsis or septic shock includes low-dose steroids for septic shock, administration of recombinant activated protein C (drotrecogin alfa), maintaining glucose control ≥70 but <150 mg/dL and maintaining median inspiratory plateau pressure (IPP) <30 cm H_2_O for mechanically ventilated patients. Hydrocortisone 300 mg/day for 7 days in 3 divided doses was administered to patients with refractory hypotension despite adequate fluid replacement and vasopressors. Activated protein C was indicated for patients with ≥2 sepsis-induced organ failures, or APACHE II score ≥25 and no contraindications.

The American College of Chest Physicians/Society of Critical Care Medicine (ACCP/SCCM) definitions were used for clinical conditions [6]. Sepsis was defined as infection plus two or more of the following SIRS criteria: T>38°C or <36°C; HR>90/min; RR>20 breaths/min (or Paco2<32 mm Hg); or WBC count, >12,000 cells/µL or <4,000 cells/µL (or >10% band forms). Severe sepsis was defined as sepsis plus organ dysfunction, hypotension, or hypoperfusion abnormalities, including lactic acidosis, oliguria, or encephalopathy. Septic shock was defined as sepsis-induced hypotension (ie, systolic BP, <90 mm Hg or a drop of >40 mm Hg in the absence of other cause of hypotension) plus hypoperfusion abnormalities despite adequate fluid resuscitation.

Our hospital has an electronic system for activating a team dedicated to diagnosing and treating severe sepsis and septic shock patients immediately. The ICU doctor and the managing nurse are simultaneously notified. The development of this sepsis team (ICU doctor and managing nurse) was part of implementing the sepsis bundle. Our hospital has also a rapid response team (named in our hospital as “code yellow”) since 2007. The “code yellow” is a new service for emergent and urgent calls. When the patient shows signs of acute alteration in their health, the code yellow is activated based on the following criteria: respiratory problems such as acute decrease in oxygen saturation <90% and change in respiratory frequency to <8/minute or >28/minute; circulatory problems: decrease in systolic arterial pressure to <90 mmHg associated with symptoms and change in heart rate to <40 bpm or >130 bpm; neurologic problems: decreasing consciousness levels and convulsions; or a serious concern with the patient's overall condition (patient claims to be feeling unwell or has the sensation “something is not right”) and change in color, diaphoresis and coolness of the patient's extremities.

### Statistical analysis

For continuous variables, mean values were compared using two sample t-tests for independent samples. Differences in proportions were compared using a Chi-square test or Fisheŕs exact test when appropriate. Mean values are reported ±1 SD. All tests of significance are two-tailed. When collinearity was identified between two variables, the one with the greatest clinical relevance associated with mortality was included in the multivariate analysis. Odds ratios were calculated for independent variables associated with in-hospital mortality in severe sepsis and septic shock patients. The association of independent variables was expressed as odds ratios with 95% confidence intervals. Alpha was set at 0.05. All statistical analyses were done using the Statistical Package for the Social Sciences software (SPSS 17.0, Chicago, IL, USA).

## Results

During the study period, a total of 564 severe sepsis and septic shock patients were identified. Patients included in this study had a mean ± standard deviation (SD) age of 66±18.7 years; fifty-seven percent were male. The mean±SD APACHE II score was 23±7.2. The organ dysfunctions at the time of diagnosis were 76.6% with cardiac dysfunction, 51.1% with respiratory dysfunction, 48.6% with renal dysfunction, 32.6% neurologic dysfunction, 28.5% with hematologic dysfunction and 10.1% with liver dysfunction. The mean±SD arterial lactate was 32±29.9 mg/dL. The mean±SD total fluid resuscitation was 2,093±1,163 mL. Septic shock and severe sepsis were present in 75.7% and 24.3%, respectively. The main source of infection was 54.6% pneumonia, 20.2% intra-abdominal infection and 14.9% urinary tract infection. The hospital setting where severe sepsis and septic shock were identified was 40.9% in emergency department, 21.6% in a medical ward, 21.5% in ICU, 9.8% in a step-down unit, 3.7% at another hospital and 2.5% in operating room. In Table 1 we divided patients in three groups, those who met the 6-hour bundle compliance (n = 69, 12.2%), those who met the 24-hour bundle compliance (but not 6-hour) (n = 124, 22.0%), and those who did not meet measure compliance for any bundle (n = 371; 65.8%).

**Table 1:** Demographic and clinical characteristics of severe sepsis and septic shock patients.

		Compliance 6 h bundle		Compliance 24 h bundle (but not 6 h)		No compliance any bundle	
		n	%	n	%	n	%
Total		69	12.2	124	22.0	371	65.8
**Age (years), mean (±SD)**		63±19.7		65±19.7		68±18.0	
**Male**		42	60.9	69	55.6	210	56.6
**Arterial lactate (mg/dL), mean (±SD)**		27±24.5		28±23.2		34±29.7	
**Apache II, mean (±SD)**		22±7.0		20±5.9		24±7.4	
**Organ dysfunction**							
Liver		2	2.9	10	8.1	45	12.1
Cardiologic		49	71.0	82	66.1	301	81.1
Renal		27	39.1	41	33.1	207	55.8
Hematologic		10	14.5	26	21.0	125	33.7
Respiratory		40	58.0	63	50.8	185	49.9
Neurologic		25	36.2	24	19.4	135	36.4
**Source of infection**							
Pneumonia		39	56.5	58	46.8	211	56.9
Intra-abdominal		15	21.7	25	20.2	74	19.9
Urinary		11	15.9	20	16.1	53	14.3
Skin/soft tissue		3	4.3	10	8.1	9	2.4
Endocarditis		0	0.0	2	1.6	6	1.6
Bloodstream infection		0	0.0	2	1.6	9	2.4
Others infections		1	1.4	7	5.6	9	2.4
**Fluids total (ml), mean (±SD)**		2269±1239		2333±1374		1971±1046	
**Previous antimicrobial therapy**		36	52.2	38	30.6	131	35.3
**Severe sepsis**		37	53.6	50	40.3	50	13.5
**Septic shock**		32	46.4	74	59.7	321	86.5
**Length of stay ICU (days), mean (±SD)**		30±141.4		9±11.3		16±62.7	
**Length of stay hospital (days), mean (±SD)**		37±81.8		38±68.4		51±122.6	
**Unit**							
Surgical room		2	2.9	4	3.2	8	2.2
Medical ward		17	24.6	22	17.7	83	22.4
Others hospital		3	4.3	4	3.2	14	3.8
Emergency department		34	49.3	66	53.2	131	35.3
Step-down unit		6	8.7	11	8.9	38	10.2
ICU		7	10.1	17	13.7	97	26.1

SD - Standard Deviation.

### Bundle compliance performance

As seen in Table 2, baseline compliance with the 6-hour bundle was only 6%. After the intervention, compliance rates were 8.2% from May to December 2006, 9.3% in 2007, 21.1% in 2008 and 13.7% in 2009. For the 24-hour management bundle, compliance was 15.0% at baseline, 15.1% from May to December 2006, 21.4% in 2007, 27.8% in 2008 and 44.4% in 2009. The distribution of diagnoses was as follows: from July 2005 to April 2006 12% were severe sepsis and 88% septic shock; from May to December 2006 15.1% were severe sepsis and 84.9% septic shock; in 2007, 22.7% were severe sepsis and 77.3% septic shock; in 2008, 24.8% were severe sepsis and 75.2% septic shock; and in 2009 41.9% were severe sepsis and 58.1% septic shock.

**Table 2:** Performance of bundle compliance.

Study phases	Before protocol	After protocol			
Period	Jul/05-Apr/06	May/06-Dec/06	2007	2008	2009
Total patients = 564	N = 100 (%)	N = 73 (%)	N = 140 (%)	N = 134 (%)	N = 117 (%)
**Severe sepsis**	12 (12.0)	11 (15.1)	32 (22.9)	33 (24.8)	49 (41.9)
**Septic shock**	88 (88.0)	62 (84.9)	108 (77.1)	100 (75.2)	68 (58.1)
Serum arterial lactate	72 (72.0)	68 (93.2)	121 (86.4)	123 (92.5)	96 (82.1)
Blood cultures prior to antibiotic administration	44 (44.0)	42 (57.5)	67 (47.9)	99 (74.4)	85 (72.6)
Broad-spectrum antibiotic within 1 hr	58 (58.0)	46 (63)	105 (75)	103 (77.4)	73 (62.4)
CVP>8 mmHg	60 (60.0)	44(60.3)	85 (60.7)	90 (67.7)	76 (65.0)
ScvO_^2^_ ≥70%	50 (50.0)	34 (46.6)	60 (42.9)	64 (48.1)	66 (56.4)
Treat hypotension (fluids plus vasopressors)	96 (96.0)	59 (80.8)	131 (93.6)	115 (86.5)	93 (79.5)
**6-hour bundle all-or-none compliance**	6 (6.0)	6 (8.2)	13 (9.3)	28 (21.1)	16 (13.7)
Median crystalloid or equivalent delivered (mL)	1667	2021	1955	2396	2401
Corticosteroids	76 (76.0)	65 (89)	84 (60.0)	112 (84.2)	117 (100)
Activated protein C administered in eligible patients	40 (40.0)	36 (49.3)	63 (45.0)	118 (88.7)	114 (97.4)
Glucose control>70≤150 mg/dL	48 (48.0)	33 (45.2)	78 (55.7)	49 (36.8)	54 (46.2)
IPP<30 cmH_^2^_O for mechanically ventilated patients	82 (82.0)	61 (83.6)	134 (95.7)	122 (91.7)	110 (94.0)
**24-hour bundle all-or-none compliance**	15 (15.0)	11 (15.1)	30 (21.4)	37 (27.8)	52 (44.4)
**Mortality rate**	54 (54.0)	30 (41.1)	55 (39.3)	55 (41.4)	19 (16.2)

CVP – Central venous pressure.

ScvO^2^ - Central venous oxygen saturation.

IPP – Inspiratory Plateau Pressure.

**Figure 1:**
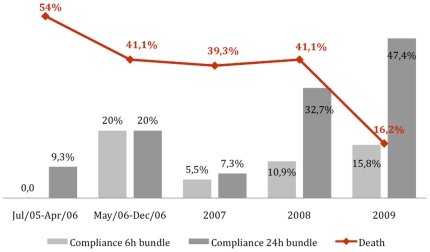
Proportion of patients with severe sepsis and septic shock who died and who had completed bundle measures during the study period.

Analyzing the resuscitation bundle (first 6 hours), the only 2 processes that showed compliance more than 65% were lactate determination and use of fluids plus vasopressors to avoid hypotension. Blood cultures collected prior to antibiotic administration improved in the last two years, 74.4% and 72.6% of compliance respectively. Regarding the management bundle (first 24 hours), the only 2 processes that showed compliance more than 65% were corticosteroids and inspiratory plateau pressure. The in-hospital mortality rates were 54.0% from July 2005 to April 2006, 41.1% from May to December 2006, 39.3% in 2007, 41.4% in 2008 and 16.2% in 2009 (Table 2). Figure 1 shows the proportion of patients with severe sepsis and septic shock who died and who had completed the bundle measures during the study period.

### 6-hour and 24-hour bundle compliance associated with mortality benefit

Univariate comparisons of mortality in severe sepsis and septic shock patients receiving the 6-hour and 24-hour sepsis bundle, and considering age, APACHE II and organ dysfunctions were performed as seen in Table 3. Variables that were statistically significant in univariate analyses were selected for multiple logistic regression. In this model, there was a statistically significant decreased odds ratio for mortality in patients who had received corticosteroids (OR 0.47; CI95 0.29–0.78, p = 0.003), and IPP<30 cmH2O for mechanically ventilated patients (OR 0.51; CI95 0.26–0.99, p = 0.047) in the 24-hour bundle.

**Table 3:** Risk factors associated with death in severe sepsis and septic shock patients.

Variables	Cases (564)		Univariate analysis		Multivariate analysis	
	Death	Survival	OR (CI_95_)	*p*	OR (CI_95_)	*p*
	N (%)	N (%)				
	213 (100)	351 (100)				
Age (years), mean (±SD)	69(±18.2)	65(±18.8)	−4.04 [(−7.19) – (−.089)]	0.012	1.02 (1.01–1.03)	0.005
Apache II, mean (±SD)	25(±7.2)	22(±6.9)	−3.47 [(−4.68) – (−2.26)]	<0.001	1.05 (1.02–1.08)	0.002
Liver dysfunction	37 (17.4)	20 (5.7)	3.47 (1.96–6.18)	<0.001	3.37 (1.76–6.44)	<0.001
Cardiologic dysfunction	181 (85.0)	251 (71.7)	2.23 (1.43–3.47)	<0.001	1.46 (0.89–2.45)	0.15
Renal dysfunction	136 (63.8)	139 (39.7)	2.68 (1.89–3.81)	<0.001	1.97 (1.32–2.96)	0.001
Hematologic dysfunction	90 (42.3)	71 (20.3)	2.88 (1.97–4.19)	<0.001	2.19 (1.40–3.41)	0.001
Respiratory dsyfunction	115 (54.0)	173 (49.4)	1.20 (0.85–1.69)	0.29		
Neurologic dysfunction	84 (39.4)	100 (28.6)	1.63 (1.14–2.33)	0.008	1.33 (0.88–2.02)	0.18
Serum arterial lactate	182 (85.4)	298 (84.9)	1.04 (0.65–1.69)	0.86		
Blood cultures prior to antibiotic administration	105 (49.3)	232 (66.1)	0.49 (0.35–0.70)	<0.001	0.69 (0.46–1.03)	0.07
Broad-spectrum antibiotic within 1 hr	149 (70)	236 (67.2)	1.13 (0.78–1.64)	0.50		
CVP>8 mmHg	131 (62.9)	224 (63.8)	0.91 (0.64–1.29)	0.58		
ScvO_^2^_ ≥70%	88 (41.3)	186 (53)	0.62 (0.44–0.88)	0.007	0.78 (0.52–1.16)	0.22
Treatment of hypotension (fluids plus vasopressors)	190 (89.2)	304 (86.6)	1.27 (0.75–2.17)	0.365		
Corticosteroids	149 (70)	305 (86.9)	0.35 (0.30–0.54)	0.003	0.47 (0.29–0.78)	0.003
Activated protein C administered in eligible patients	108 (50.7)	263 (74.9)	0.35 (0.24–0.50)	<0.001	0.92 (0.58–1.48)	0.74
Glucose control >70 but ≤150	89 (41.8)	173 (49.3)	0.74 (0.52–1.04)	0.083		
IPP<30 cmH_^2^_O for mechanically ventilated patient	181 (85)	328 (93.4)	0.40 (0.22–0.70)	<0.001	0.51 (0.26–0.99)	0.047

CVP – Central venous pressure.

ScvO^2^ - Central venous oxygen saturation.

IPP – Inspiratory Plateau Pressure.

There was also a statistically significant ratio for mortality in older patients (OR 1.02; CI95 1.01–1.03, p = 0.005), a higher APACHE II score (OR 1.05; CI95 1.02–1.08, p = 0.002), liver dysfunction (OR 3.37; CI95 1.76–6.44, p<0.001), renal dysfunction (OR 1.97; CI95 1.32–2.96, p = 0.001), and hematologic dysfunction (OR 2.19; CI95 1.40–3.41, p = 0.001).

A statistically significant decreased odds ratio for mortality in patients was observed when there was complete compliance with the 6-hour bundle (OR 0.54; CI95 0.30–0.96, p = 0.033) and when there was complete compliance with all the indicated components of the 24-hour bundle (OR 0.37; CI95 0.24–0.58, p<0.001).

## Discussion

This study demonstrates that implementing a sepsis bundle improved the outcomes of patients with severe sepsis and septic shock. [7]. At the beginning of the study, it was not easy to convince physicians that it is necessary to apply these simple measures and our compliance was not more than fifty percent for the 24-hour bundle.

Something needed to be done since our mortality rates for severe sepsis and septic shock were extremely high. Our hospital is engaged in a patient safety program that is a resource from the IHI. The implementation of a sepsis response team available across the hospital allowed healthcare workers to call the sepsis team (based in the ICU) for all suspected sepsis cases. In addition, the implementation of the rapid response team contributed to decreasing our mortality rates from 52% in 2005 to 16% in 2009.

Our study differs from the other studies that applied the sepsis bundles only in the emergency department as a quality indicator set to modify physician behavior related to the early management of severe sepsis and septic shock [8], [9], [10], [11]. Similar to the others we implemented the sepsis bundle not only in the emergency department, but also in medical and surgical wards. All ICU patients were actively screened daily for the presence of severe sepsis or septic shock [12], [13], [14]. The implementation of the Surviving Sepsis Campaign guidelines was associated with a significant decrease in mortality. In using these guidelines no extra staff were allocated, but we assigned an ICU doctor and an ICU nurse to be responsible for the sepsis bundle process during their ICU duty.

Even though the sepsis bundles showed a real benefit for decreasing mortality from severe sepsis and septic shock, there is a considerable gap between the science and its application [15]. A long period often exists between initial experimental results and their transformation into new technologies in health. From bench to bedside there is a difficult translation from clinical trial into practice corroborated by the low sepsis bundle compliance demonstrated in other studies [8], [13], [14]. Our data are similar to these studies with a lower compliance to the bundle sepsis (<50% of compliance in the 6-, 24-hour sepsis bundle). We believe that the barriers faced to the sepsis bundle compliance are similar in other protocols (also considering the learning curve of sepsis knowledge for all HCWs in our ICU).

Our data did not show what are the most important interventions with impact on mortality in the 6-hour bundle. We do not have data about antimicrobial therapy adequacy because it is not a component of the Surviving Sepsis Campaign. Although there is a requirement for early antibiotic administration, there is no requirement for early administration of appropriate antimicrobials. Many studies have demonstrated that inadequate antibiotic therapy is related to an increase in the mortality rate [16], [17], [18]. Considering the achievement of SVO_2_>70%, an Australasian multicenter study [10] had an ICU and overall in-hospital mortality of 18.8% and 23.1% without including SVO_2_-directed resuscitation in the sepsis bundle protocol. On the other hand, another multicenter study showed that the only intervention from the sepsis bundle with impact on mortality was the achievement of SVO_2_>70% [13].

In our study, patients who had received corticosteroids (OR 0.47; CI95 0.29–0.78, p = 0.003), and IPP<30 cmH2O for mechanically ventilated patients (OR 0.51; CI95 0.26–0.99, p = 0.047) in the 24-hour bundle compliance had a better outcome. It is important to mention that activated protein C was not associated with a lower mortality in our patients and in 2009 only two patients met criteria for receiving activated protein C (APACHE II score ≥25, ≥2 sepsis-induced organ failure and no contraindications), thus 113 patients in 2009 had no indications for receiving activated protein C. This brings into question of the role of activated protein C. Over the course of the study the proportion of study patients with septic shock decreased from 88% to 58%. We believe that the implementation of the sepsis bundle prompted the ICU team to identify more patients with early sepsis and to implement a more specific treatment.

There are several limitations to this study. This is not a randomized trial but a quasi-experimental, interrupted time series study. Quasi-experimental study designs are frequently used when it is not logistically feasible to conduct a controlled trial. Thus, other unmeasured factors might have occurred coincident with the interventions that occurred since May 2006 (implementation of the sepsis bundle), resulting in a decrease in severe sepsis and septic shock mortality in our hospital. Finally, because this intervention was performed at a single medical center, these results may not be generalizable to other hospitals. Despite the limitations, our study has broadened support for the concept that severe sepsis and septic shock patients require multiple performance measures and quality improvement efforts to improve outcomes. The process and outcome measures for septic patients presented here are derived from published guidelines and other relevant literature.

We believe that the fall in mortality is attributed to the better care of the sepsis patient by applying the sepsis bundle and to identify the sepsis patients. Since 2007 our hospital has been engaged in zero tolerance for healthcare associated infections. We have observed a significant reduction in ventilator-associated pneumonia and in central venous associated bloodstream infections [19], [20]. We have also during the study period implementing other ICU best practices, including glycemic control protocol [21]. We adopt an intermediate glucose control, because we believe that a tight glucose control is difficult to accomplish in routine intensive care unit settings and is associated with a significant increase in the incidence of hypoglycaemia [21], [22]. However it is interesting to note that 41% of our patients included in the study are from the emergency department; this affirms our belief that the sepsis bundle needs to be considered as the intervention decreasing mortality in septic shock and severe sepsis because of the better care and the prompt recognition of these patients in the emergency room.

In conclusion, the sepsis bundle is a quality improvement program that should be implemented in all hospital settings, and efforts should be made to improve bundle compliance. Further understanding of the importance of the components of the bundle is needed to determine which components can be changed or replaced.
